# The zinc finger domain of Wilms' tumor 1 suppressor gene (WT1) behaves as a dominant negative, leading to abrogation of WT1 oncogenic potential in breast cancer cells

**DOI:** 10.1186/bcr1743

**Published:** 2007-07-16

**Authors:** Youqi Han, Serban San-Marina, Lin Yang, Haytham Khoury, Mark D Minden

**Affiliations:** 1 Department of Cellular and Molecular Biology, Ontario Cancer Institute, University Health Network, Toronto, Ontario M5G 2M9, Canada

## Abstract

**Introduction:**

There is growing evidence that the Wilms' tumor 1 suppressor gene (WT1) behaves as an oncogene in some forms of breast cancer. Previous studies have demonstrated that the N-terminal domain of WT1 can act as a dominant negative through self-association. In the studies presented here we have explored the potential for the zinc finger domain (ZF) of WT1 to also have dominant-negative effects, and thus further our understanding of this protein.

**Methods:**

Using full-length and ZF-only forms of WT1 we assessed their effect on the WT1 and c-*myc *promoter using luciferase and chromatin immunoprecipitation assays. The gene expression levels were determined by quantitative real-time RT-PCR, northern blot and western blot. We also assessed the effect of the ZF-only form on the growth of breast cancer cell lines in culture.

**Results:**

Transfection with WT1–ZF plasmids resulted in a stronger inhibition of WT1 promoter than full-length WT1 in breast cancer cells. The WT1–ZF form lacking the lysine–threonine–serine (KTS) insert (ZF - KTS) can bind to the majority of WT1 consensus sites throughout the WT1 promoter region, while the ZF containing the insert (ZF + KTS) form only binds to sites in the proximal promoter. The abundances of endogenous WT1 mRNA and protein were markedly decreased following the stable expression of ZF - KTS in breast cancer cells. The expressions of WT1 target genes, including c-*myc*, Bcl-2, amphiregulin and TERT, were similarly suppressed by ZF - KTS. Moreover, WT1–ZF - KTS abrogated the transcriptional activation of c-*myc *mediated by all four predominant isoforms of WT1 (including or lacking alternatively spliced exons 5 and 9). Finally, WT1–ZF - KTS inhibited colony formation and cell division, but induced apoptosis in MCF-7 cells.

**Conclusion:**

Our observations strongly argue that the WT1–ZF plasmid behaves as a dominant-negative regulator of the endogenous WT1 in breast cancer cells. The inhibition on proliferation of breast cancer cells by WT1–ZF - KTS provides a potential candidate of gene therapy for breast cancer.

## Introduction

The Wilms' tumor 1 suppressor gene (WT1) was first identified as a mutated gene in some cases of sporadic Wilms' tumor, a malignancy of the kidney affecting pluripotent embryonic renal precursor cells [1]. As the gene is occasionally mutated in the sporadic form of Wilms' tumor, it was assumed that WT1 was a tumor suppressor and that the mutations abrogated that function [2,3]. More recent data showing that wild-type WT1 is expressed in the majority of Wilms' tumors, and that the wild-type form is expressed in a variety of cancers including breast cancer, renal cell cancer, ovarian cancer, mesothelioma cancer, lung cancer, melanoma and acute leukemia, however, have implicated WT1 as an oncogene in those tumors [4-9]. This implication is strengthened by gene transfer and gene inhibition studies. High levels of WT1 are also associated with poor prognosis in both breast cancer and leukemia [10-12].

WT1 is a 449-amino-acid protein (molecular weight 52~54 kDa), with a glutamine and proline-rich N-terminal domain involved in transcriptional repression and activation, and with a C-terminal domain composed of four Cys–Cys–His–His-type zinc finger domains (ZFs) – which are involved in DNA and RNA binding and protein–protein interactions [13,14]. Although there are multiple forms of WT1, there are four main ones designated isoform A, isoform B, isoform C and isoform D. These isoforms are generated through two alternative splicing events: one involves the 17 amino acids of exon 5 just ahead of the ZF; the other alternative splicing event occurs in exon 9 and results in the insertion of three amino acids, lysine–threonine–serine (KTS), between the third and fourth ZF [15]. The WT1 - KTS form, lacking the KTS insert, has been shown to bind GC-rich DNA sequences such as the WTE site (5'-GCGTGGGAGT-3') [16], the more degenerate 5'-GNGNGGGNG-3' motif [17], or the (TCC)_*n *_motif [18]. There is considerable overlap between the known targets of WT1 and those of early growth response protein 1; this is in keeping with the high degree of homology between the ZF of WT1 and early growth response protein 1 [13].

Several transcription factors have been shown to regulate the WT1 promoter using reporter assays, including PAX2 [19,20], PAX8 [21,22], early growth response protein 1 [23] and SP1 [24]. Of particular note is the autorepression of the mouse WT1 promoter by full-length WT1 [25]. These latter experiments were performed in HEK-293 cells. In addition, a number of other genes have been shown to be direct targets of WT1 [17]. Among these genes are epidermal growth factor receptor, c-*myc*, and Bcl-2. For many of these genes it has been suggested that WT1 behaves as a repressor of transcription. Our studies and those of other workers, however, have found that WT1 behaving as a repressor or as an activator is dependent upon the cellular context [26,27]. For example, in Hela cells, WT1 inhibits the transactivation of c-*myc *– while in the breast cancer cell line MDA468, WT1 activates c-*myc *expression. Regardless of whether WT1 is behaving as a repressor of or as an activator of gene expression, this activity is due to the direct binding of the ZF of WT1 to DNA. In the current study we have explored the ability of WT1–ZF to act as a dominant negative. In approaching this goal we first demonstrate that WT1 itself is regulated by ZF domain of WT1, and that the endogenous expression of WT1 can be inhibited by WT1–ZF. We then go on to demonstrate in WT1-expressing breast cancer cells that WT1–ZF behaves as a dominant negative and inhibits cell growth and survival.

## Materials and methods

### Cell culture

The human breast cancer lines MCF-7 (ATCC catalogue number HTB22), MDA468 (ATCC catalogue number HTB132) and MDA231 (ATCC catalogue number HTB26) were routinely subcultured in minimal Eagle's medium supplemented with 10% heat-inactivated fetal bovine serum and antibiotics in a humidified atmosphere of 5% CO_2 _at 37°C. The breast cancer cells overexpressing WT1 were cultured in media supplemented with 0.5 mg/ml G418.

### Plasmids

The expression plasmids for the four full length murine WT1 isoforms (A, B, C, D), aminoterminal-only construct N-WT1 (amino acid residues 1–180), WT1–ZF - KTS or WT1–ZF + KTS (amino acid residues 318–449), were constructed by cloning the corresponding coding region into pcDNA_3 _vector (Invitrogen, Carlsbad, CA, USA). The plasmids for expression of the GST/WT1–ZF - KTS and the GST/WT1–ZF + KTS fusion proteins in *Escherichia coli *were generated by cloning the fragments encoding the above WT1–ZF constructs into *Sma*I/*Eco*RI-digested pGEX2TK (Pharmacia, North Peapack, NJ, USA), in frame with the GST domain.

To generate plasmid expressing WT1 shRNA, double-stranded oligonucleotides were cloned into the *Hind *III/*Bgl *II sites in pSuper vector (Oligoengine Inc., Seattle, WA, USA). The WT1 shRNA sequence used is gatccccTCAGGGTTACAGCACGGTCttcaagagaGACCGTGCTGTAACCCTGAtttttgga (uppercase letters represent WT1-specific sequence, and lowercase letters represent hairpin sequences).

The c-*myc *reporter constructs XNM/Luc and Xmut/Luc have been previously described [27]. The promoter constructs for human hW3/Luc (nucleotides -443 to +21) and mouse mW2/Luc (nucleotides -502 to +21) were generated by PCR amplification of the corresponding promoter regions and cloning into the *Sac*I/*Xho*I sites of the pGL2 enhancer vector (Promega, Madison, WI, USA). Promoter constructs for hW10/Luc (nucleotides -443 to +182) and mW9/Luc (nucleotides -502 to +181) were cloned into the *Kpn*I/*Bgl*II sites of the pGL2 enhancer vector.

All constructs were verified by restriction enzyme digestion or by sequencing.

### Transfection and luciferase assays

Triplicate transfections in 60 mm plates were performed using Lipofectamine (Invitrogen) and the following transfection mix: 1 μg luciferase reporter (pGL2 vectors as control), 0.1 μg β-galactosidase internal control vector and 3 μg WT1 expression plasmid (pcDNA_3 _containing no insert as control). After 48 hours the cells were washed twice with PBS and were harvested for luciferase and β-galactosidase assays, which were performed according to the manufacturer's protocols (Promega). Promoter activation in cells transfected with promoter constructs, relative to cells transfected with pGL2, was calculated by dividing luciferase activities normalized to β-galactosidase expression between the two transfections. The results of all assays are the average of at least three experiments.

To obtain stable expression of WT1 proteins, cells were transfected with expression constructs or the empty pcDNA_3 _vector that contains a G418 resistance gene, and were then cultured in selective medium containing 0.5 mg/ml G418 for at least 2 weeks. Several colonies from the transfected cell pool were isolated and expanded. Overexpression of WT1 protein was determined by western blotting.

### Chromatin immunoprecipitation and PCR amplification

Chromatin immunoprecipitation reactions were performed using the Chromatin Immunoprecipitation Assay Kit (Upstate Biotech, Charlottesville, VA, USA) modified as follows. Approximately 1 × 10^7 ^cells were cross-linked with 1% formaldehyde at 37°C for 15 minutes, and the cross-linking reaction was terminated with 0.125 M glycine at room temperature for 10 minutes. Cells were washed in PBS, resuspended in 200 μl SDS lysis buffer (1% SDS, 10 mM ethylenediamine teraacetic acid, 50 mM Tris–HCl, pH 8.1) and incubated on ice for 15 minutes. Chromatin was sonicated on ice to an average length of 500 bp using a Fisher Scientific Dismembranator 100 with 7 × 15 second pulses at a 4 W power output setting. Cell debris was removed by centrifugation at 12,000 × *g *for 15 minutes at 4°C. The sheared chromatin was precleared with salmon sperm DNA/protein A agarose for 30 minutes at 4°C and was subjected to immunoprecipitation with 2 μg either anti-WT1 (C-19) (Santa Cruz Biotech, Santa Cruz, CA, USA), anti-HA (Roche, Grenzacherstrasse, Basel, Switzerland) or anti-acetyl-histone H4 antibody (Upstate Biotech) overnight at 4°C. A no-chromatin mock control and a preimmune serum control were prepared in parallel. One-tenth of the supernatant obtained following immunoprecipitation with preimmune serum sample was used as the chromatin input. After incubated with the secondary antibody for the additional 1 hour at 4°C, followed by incubation with salmon sperm DNA/protein A agarose for 30 minutes, the immunoprecipitated chromatin complexes were washed five times according to the manufacturer's protocol and were eluted with the elution buffer (50 mM NaHCO_3 _and 1% SDS). Cross-links were reversed with 0.3 M NaCl for 4 hours at 65°C, proteins were digested with proteinase K for 2 hours at 45°C, and DNA was extracted with phenol/chloroform, precipitated with ethanol and resuspended in 30 μl water.

PCR reactions containing 3 μl of the above DNA preparation, primers and Platinum Taq (Invitrogen) in a 50 μl final volume was performed with the initial denaturation at 95°C for 4 minutes, followed by 35 PCR cycles (95°C for 45 seconds, 55°C for 30 seconds and 68°C for 1 minute) and a final extension at 72°C for 10 minutes. The sequences of the forward and reverse primers for amplification of the endogenous human WT1 promoter were: P1 forward, 5'-GGTTGAAGAGGAGGGTGTCTC-3' and P1 reverse, 5'-GCTTCCCAAAGCTCAAATAAG-3'; and P2 forward, 5'-CAGCTGGGGTAAGGAGTTCAAG-3' and P2 reverse, 5'-CAAGAGGAAGTCCAGGATCGC-3'. The sequences of primers for human aldehyde dehydrogenase 1 family member A2 (ALDH) promoter were: ALDH forward, 5'-CAATTTCATACATAGGGAGACCAAG-3'; and ALDH reverse, 5'-TAGGCTATCTTGCAGAGCCAAA-3'. The specificity of the PCR amplification reaction was determined by sequencing the correct-sized bands extracted from agarose gels.

### Electrophoretic mobility shift assays

The purified GST fusion proteins (1 μg) were incubated with 40,000 cpm ^32^P-labeled WT1 binding site wild-type double-stranded oligonucleotide and with 1 μg poly(dA–dT) in a buffer containing 8% glycerol, 100 mM NaCl, 5 mM MgCl_2_, 5 mM dithiothreitol and 0.1 mg/ml phenylmethylsulphonyl fluoride in a final volume of 20 μl for 15 minutes at room temperature. DNA-bound protein complexes were separated by electrophoresis. The sequence of wild-type oligonucleotide probe was 5'-CG**CTCCCCCAC**TTCC**CGCCCTCCCTCCC**ACCTACTCATT**CACCCACCCACCCACCC**AGAG-3'. The sequence of the mutant probe was 5'-CG**ATCATAGAT**TTCC**ATATATGACTATT**ACCTACTCATT**GAATAATGAGTATTATG**AGAG-3' (WT1 binding sites are boldfaced and underlined). These sites were all changed in the oligonucleotide mutant probe.

### Quantitative real-time RT-PCR

RNA was extracted with the RNeasy kit (Qiagen, Mississauga, Ontario, Canada) and converted into cDNA with RT (Invitrogen). PCR reactions containing SYBR Green Master Mix (Applied Biosystems, Foster City, CA, USA), 100 nM primer and template cDNA in a 25 μl final volume were performed in the ABI PRISM 7799 thermocycler (Applied Biosystems) with the following parameters: an initial denaturation at 95°C for 10 minutes, followed by 40 PCR cycles (95°C for 15 seconds, 60°C for 60 seconds) with the continual measurement of fluorescence.

The primer sequences for gene amplification were as follows. GAPDH forward, 5'-GAAGGTGAAGGTCGGAGTC-3' and GAPDH reverse, 5'-GAAGATGGTGATGGGATTTC-3'; hWT1 forward, 5'-GAGAGCCAGCCCGCTATTC-3' and hWT1 reverse, 5'-CATGGGATCCTCATGCTTG-3'; mWT1 forward, 5'-TCAAGGACTGCGAGAGAAGG-3' and mWT1 reverse, 5'-TGGTGTGGGTCTTCAGATGG-3'; c-*myc *forward, 5'-AGGCCCCCAAGGTAGTTATCC-3' and c-*myc *reverse, 5'-TTTCCGCAACAAGTCCTCTTC-3'; Bcl-2 forward, 5'-CCTGTGGATGACTGAGTACCTGAAC-3' and Bcl-2 reverse, 5'-GGCCAAACTGAGCAGAGTCTTC-3'; amphiregulin (AREG) forward, 5'-GGTGGTGCTGTCGCTCTTG-3' and AREG reverse, 5'-GGTCCCCAGAAAATGGTTCA-3'; TERT forward, '-GTCTTGCGGCTGAAGTGTCA-3' and TERT reverse, 5'-TCCAAACTTGCTGATGAAATGG-3'; and brain acid soluble protein 1 (BASP1) forward, 5'-GGCACCGCGCTAACTCA-3' and BASP1 reverse, 5'-TTGGCTTTCTCGTCGTTCAC-3'.

Specificity of the PCR amplification was demonstrated by no amplification of the samples lacking a template, and by sequencing of amplified templates. The calculation of relative expression was performed as described by the manufacturer (PE Applied Biosystems), using the comparative threshold cycle normalized to GAPDH. Results are shown as the mean ± standard deviation from three independent experiments.

### Northern blotting

Fifty micrograms of total cellular RNA was extracted using Trizol, was fractionated on agarose–formaldehyde gels and was transferred by capillary suction to nylon membranes. Blots were prehybridized for 4 hours in hybridization buffer (0.5 M sodium phosphate, pH 7.2,7% SDS, 1 mM ethylenediamine tetraacetic acid, 1% bovine serum albumin) at 65°C. The membrane was then incubated with 1 × 10^6 ^cpm ^32^P-WT1 cDNA probe per milliliter overnight in the same buffer, washed three times with 1% SDS-containing phosphate buffer at 65°C, and was exposed to X-AR film for 24–48 hours. For the β-actin RNA hybridization, the same membrane was reprobed with ^32^P-labeled β-actin cDNA and was briefly exposed.

### Western blotting

Two hundred micrograms of whole cell extracts was boiled in Laemmli buffer, was separated on a 10% SDS-PAGE, and was transferred to polyvinylidene difluoride membranes (Millipore, Billerica, MA, USA). Membranes were then blocked in PBS containing 8% milk and were immunoblotted with either affinity-purified rabbit polyclonal anti WT1 (C-19) antibody (Santa Cruz Biotech), which recognizes residues 431–449 of WT1, or with rabbit anti-β-actin antibody (Sigma, Oakville, Ontario, Canada). Immune complexes were detected by binding anti-rabbit IgG conjugated to horseradish peroxidase (Santa Cruz Biotech) followed by reaction in the enhanced chemiluminescence assay (Amersham Bioscience, Piscataway, NJ, USA) according to the manufacturer's recommendations.

### Colony assay

Cells were plated in 6 cm dishes at a density of 5 × 10^5 ^per plate and were transfected with 2 μg either control or expression plasmid. After 48 hours the cells were trypsinized and plated into five 10-cm dishes for each transfectant. After cells were selected in medium containing 0.5 mg/ml G418 for 2 weeks, the numbers of G418-resistant colonies were counted. The assay results are the average of three individual experiments.

### Cell division assay

MCF-7 cells (~2 × 10^6 ^to 5 × 10^6^) were transfected with 10 μg either control or expression plasmid per 10 cm plate and cultured at 37°C for 48 hours. Following washing twice with PBS, cells were labeled with 5 μM carboxy-fluorescein diacetate succinimidyl ester (CFSE) (Molecular Probes, Eugene, OR, USA) at 37°C for 20 min. After the CFSE was washed out, cells were selected in the medium containing 0.5 mg/ml G418 for the indicated time. The CFSE-labeled transfectants were then analyzed by a FACScan flow cytometer (Becton Dickinson, Franklin Lakes, NJ, USA) using excitation at 488 nm and the FL1 channel. At least 50,000 events were collected in each final gated histogram.

The CFSE profiles were determined using Cell Quest software (Becton Dickinson). The difference of average division numbers between the two kinds of cell populations was calculated according to the following formula: *N*_1 _- *N*_2 _= (lg *A*_2 _- lg *A*_1_)/lg 2, where *N*_1 _and *N*_2 _are the average division numbers in faster-growing and slower-growing cell populations, and *A*_1 _and *A*_2 _are the mean fluorescence intensities of faster-growing and slower-growing cell populations labeled with CFSE, respectively.

### Apoptotic assay

MCF-7 cells were transfected with either control or expression plasmid for 48 hours and were then cultured in the selective medium for the indicated time. The transfectants of MCF-7 cells were stained with Annexin V-PE and 7-amino-actinomycin D (7-AAD) (BD Pharmingen, San Diego, CA, USA) according to the manufacturer's recommendation. Appropriate gates of the intact cells, based on individual controls, were utilized to determine the percentages of the cell population at the earlier stage (Annexin V positive, 7-AAD negative) and at the later stage (Annexin V positive, 7-AAD positive) of apoptosis.

## Results

### WT1–ZF abrogates transcriptional activity of the WT1 promoter

**Figure 1:**
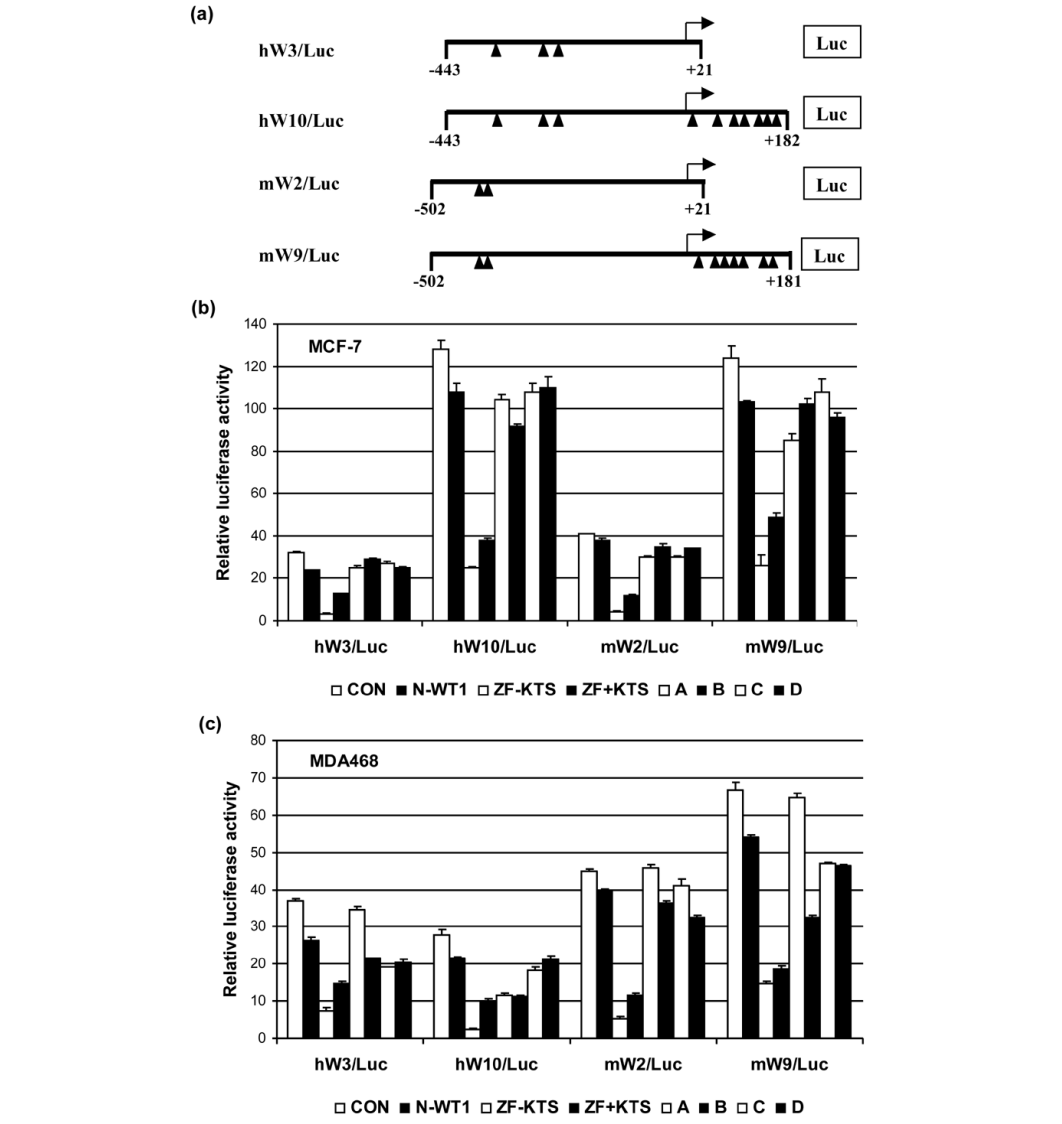
Inhibition of transcriptional activity of the WT1 promoter by its zinc finger domain. **(a) **Schematic drawing for luciferase reporter constructs. The predominant transcription start site (+1) is indicated by an arrow. Small solid triangles indicate Wilms' tumor 1 suppressor gene (WT1) binding sites. **(b) **MCF-7 cells and **(c) **MDA468 cells were co-transfected with plasmids expressing the WT1 proteins (either pcDNA_3 _control (CON), aminoterminal-only construct N-WT1, zinc finger domain lacking or with lysine–threonine–serine (ZF - KTS or ZF + KTS) or full-length WT1 vectors A~D), and either the pGL2 vector control or the differential WT1 promoter-driven luciferase constructs (horizontal axis), respectively. Luciferase activity was normalized with β-galactosidase activity and is expressed in relative luciferase activity as compared with the luciferase vector control (vertical axis). Results are the average of three experiments.

In our search to identify genes regulated by WT1, we identified two regions in the human WT1 promoter that contain WT1 binding sites and are conserved between mouse and human. To investigate the effect of full-length WT1 and the WT1–ZF constructs on the human and mouse WT1 promoter, varying lengths of the human and mouse genes were cloned upstream of firefly luciferase in the pGL2 vector (Figure 1a). The position of the WT1 binding sites in the promoters is indicated. The human hW3/Luc and mouse mW2/Luc overlap the WT1 minimal promoter located from -202 to -99 in the human promoter [28]. The hW10/Luc and mW9/Luc constructs contain the full-length promoter of WT1 [29,30].

These constructs were then co-transfected with either full-length WT1 or WT1–ZF expression plasmids into the WT1-expressing breast cancer cells MCF-7 or MDA468. In contrast to the empty vector or full-length WT1, the WT1–ZF constructs caused a dramatic suppression of luciferase activity regardless of the cell line or the presence or absence of the KTS insert. It is of note that the ZF - KTS form was more suppressive than the ZF + KTS form (Figure 1b,c). We also included an aminoterminal-only construct (N-WT1) that contains amino acids 1–190 as this has previously been shown to repress transcription of WT1 targets [31-33]. In the case of the WT1 promoter, N-WT1 had significantly less suppressive effect when compared with the ZF constructs.

### WT1–ZF binds to the WT1 promoter *in vivo *and *in vitro*

**Figure 2:**
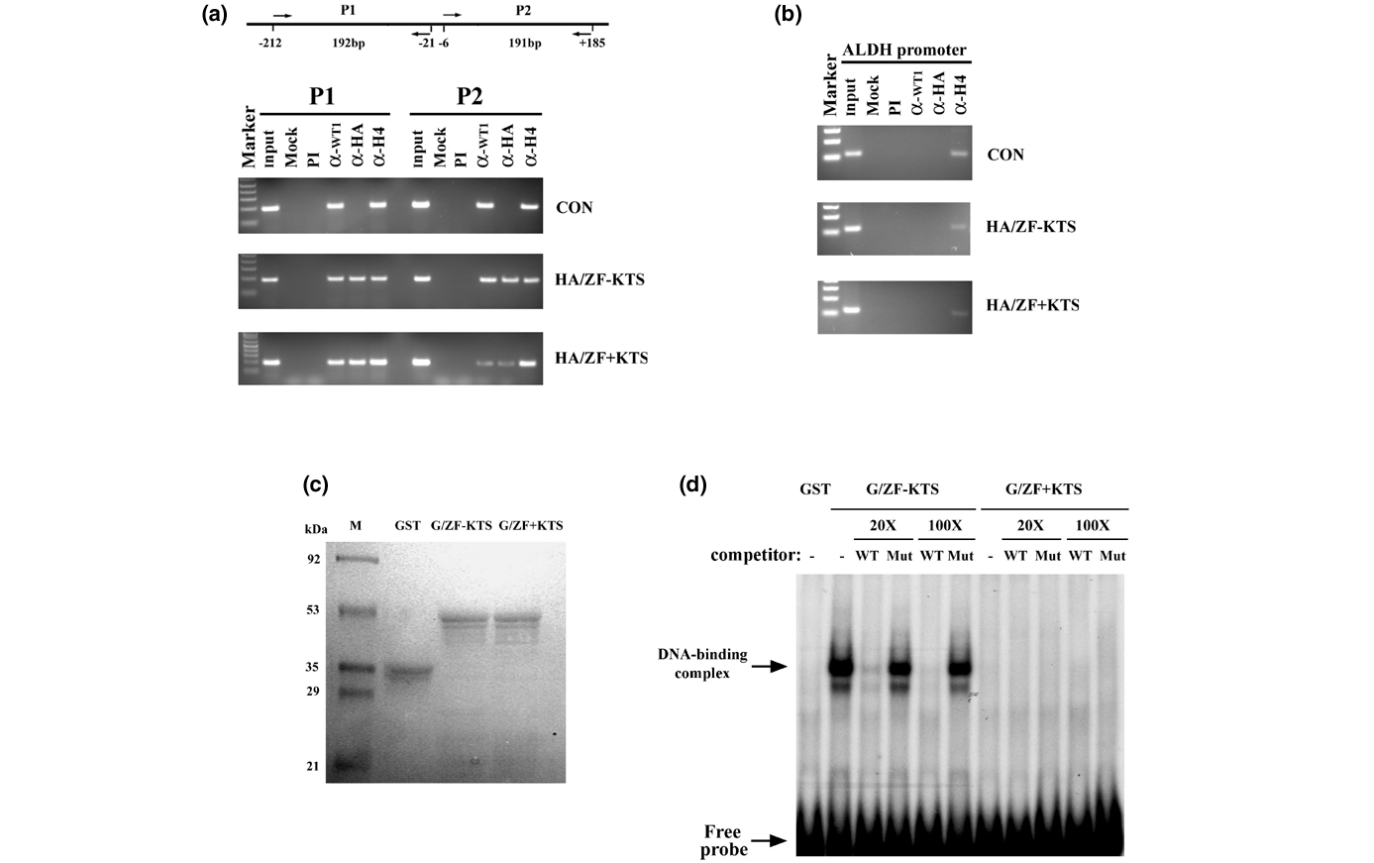
WT1–ZF protein specific binding to WT1 consensus motifs in WT1 promoter *in vivo*/*in vitro*. **(a) **Chromatin immunoprecipitation/PCR analysis for the cross-linked sheared chromatin from control MCF-7 cells (CON) and MCF-7 cells expressing HA-tagged zinc finger domain (HA/ZF-KTS, HA/ZF+KTS) of Wilms' tumor 1 suppressor gene zinc (indicated on right). Upper panel, schematic representation of PCR-amplified fragments P1 and P2 in endogenous human WT1 promoter. Input, mock control and immunoprecipitation with preimmune serum (PI), anti-WT1, anti-HA or anti-acetyl-histone H4 antibody are indicated. Left lane, 1 kb DNA ladder as marker. **(b) **Same chromatin immunoprecipitation/PCR analysis as (a) with aldehyde dehydrogenase 1 family member A2 (ALDH) promoter primers. **(c) **Coomassie blue-stained gel. The bacteria-expressed GST and GST/WT1–ZF fusion proteins (shown as GST, G/ZF-KTS and G/ZF+KTS) were purified, fractionated in a 10% SDS-PAGE and visualized by Coomassie blue stain. The molecular sizes of standard protein markers (M) are shown. **(d) **Competition electrophoretic mobility shift assays were performed using either GST only or GST/WT1–ZF fusion proteins (G/ZF-KTS, G/ZF+KTS) with the ^32^P-labeled oligonucleotide of human WT1 proximal promoter in the absence (-) or presence of 20-fold or 100-fold molar excess of WT1 binding site wild-type or site mutant cold oligonucleotides (WT, Mut) as indicated. Migration of DNA-binding complexes induced by GST/ZF-KTS protein and free probe is shown by an arrow. The lane GST displayed no DNA-binding associated with purified GST protein. KTS, lysine–threonine–serine.

To determine whether WT1–ZF binds to the endogenous human WT1 promoter, we introduced HA tagged WT1–ZF into WT1-expressing MCF-7 cells, and selected stable transformants. Using antibody against the HA tag or acetylated histone H4 as a control, we performed chromatin immunoprecipitation followed by PCR amplification of DNA with primers specific for the P1 and P2 regions of the WT1 promoter (Figure 2a). Both regions contain several potential WT1 binding sites (Figure 1a). In the control cell line, P1 and P2 amplifiable fragments were immunoprecipitated with antibody against WT1 but not with an anti-HA antibody. In cells expressing HA-tagged WT1–ZF, the P1 and P2 fragments were precipitated with anti-WT1 and anti-HA. The ALDH promoter, however, which does not contain a WT1 binding site, was not precipitated with these antibodies (Figure 2b).

Combined, these studies show that wild-type WT1 and the ZF-only versions can bind to the WT1 promoter. For the ZF - KTS version, approximately equal amounts of P1 and P2 were amplified following chromatin immunoprecipitation with anti-HA. For the ZF + KTS form, however, much less P2 was precipitated. As the method is semiquantitative, the different intensities between the amounts of P1 and P2 precipitated by ZF + KTS may reflect differences in binding affinity or may reflect technical variation. For this reason we used electrophoretic mobility shift assays to further investigate the binding of the ZF to the P2 region of the WT1 proximal promoter. Purified GST/ZF fusion proteins produced and isolated from *E. coli *were incubated with radiolabeled oligonucleotides extending from +61 to +120 of the human WT1 promoter (Figure 2c,d). Under the conditions used, only the ZF - KTS form bound the P2 oligonucleotide. The specificity of the reaction was demonstrated by competition with excess cold wild-type but not with mutant P2 oligonucleotide. Taken together, these studies show that the ZF - KTS form binds to the internal +61 to +120 region.

### Endogenous WT1 expression is downregulated by ZF - KTS

The above results indicate that WT1–ZF is a strong repressor of an exogenous WT1 promoter in the context of the breast cancer cell lines MCF-7 and MDA468. As the ZF - KTS form is more repressive than the ZF + KTS form, experiments designed to show inhibition of the endogenous WT1 expression were carried out only with the ZF - KTS form.

**Figure 3:**
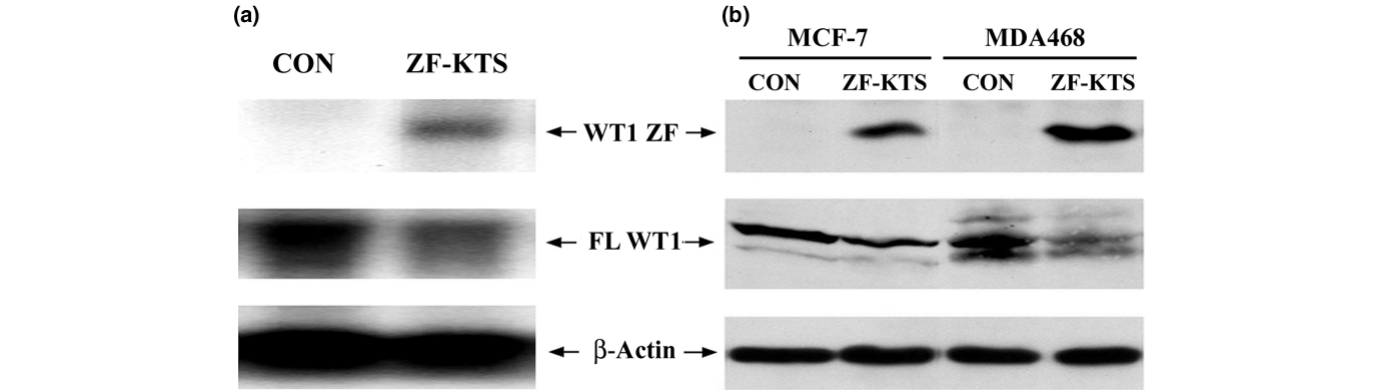
Decrease of endogenous WT1 abundance by overexpression of zinc finger domain in breast cancer cells. **(a) **Northern blot: total RNAs were isolated from the stable transfectants of MCF-7 cells with either pcDNA3 control (CON) or the zinc finger domain lacking the lysine–threonine–serine insert (ZF - KTS) of Wilms' tumor 1 suppressor gene (WT1) expression plasmids as indicated, and were analyzed with WT1 and β-actin probes. Overexpressed WT1–ZF, endogenous WT1 (FL WT1) or β-actin RNA is indicated. **(b) **Western immunoblot: whole cell extracts were prepared from the stable transfectants of MCF-7 cells or MDA468 cells with either pcDNA_3 _control or ZF - KTS expression plasmids, and were analyzed by western blot with either anti-WT1 and β-actin antibodies. Overexpressed WT1–ZF, endogenous WT1 (FL WT1) and β-actin proteins are indicated.

The MCF-7 and MDA468 cells were transfected with ZF - KTS and stable clones were isolated. To assess the level of expression of the endogenous gene and the transfected mouse gene, we used quantitative real-time PCR with primers to amplify nucleotides 439–541 of human WT1 (detects endogenous gene only) and nucleotides 1,071–1,207 of mouse WT1 (detects the transfected gene), respectively. We found that the mRNA levels of the ZF - KTS in the stable transfectants were 395-fold and 1,894-fold higher than in control MCF-7 and MDA468 cells, respectively. In contrast, the endogenous WT1 gene was reduced by 43% (MCF-7) and by 28% (MDA468) in WT1–ZF-transfected cells as compared with nontransfected control cells (Table 1). The decreased RNA levels were confirmed on northern blot analysis (Figure 3a). In keeping with the reduced levels of RNA, there was a decrease in endogenous WT1 protein (Figure 3b). These findings show that the WT1–ZF - KTS can repress the endogenous WT1 expression in breast cancer cell lines.

**Table 1:** Quantitative Wilms' tumor 1 suppressor gene (WT1) expression in stable transfectant of breast cancer cells with quantitative real-time PCR

	MCF-7 cells		MDA468 cells	
	
Transfected plasmid	Control	ZF - KTS	Control	ZF - KTS
Mouse WT1–ZF	1 ± 0.2	395 ± 66	1 ± 0.2	1894 ± 136
Human WT1	1 ± 0.1	0.43 ± 0.07	1 ± 0.2	0.28 ± 0.16

ZF - KTS, zinc finger domain lacking the lysine–threonine–serine insert of WT1.

### Several WT1 target genes are downregulated by ZF - KTS

Having shown that the ZF - KTS can decrease WT1 expression itself, we went on to determine whether other known WT1 target genes were similarly affected. Using quantitative real-time PCR we assessed the level of expression of c-*myc*, Bcl-2, AREG, TERT and BASP1 in WT1–ZF and in control cells on days 4, 8, and 12 after transfection. BASP1 was included as a non-WT1 target gene control.

**Figure 4:**
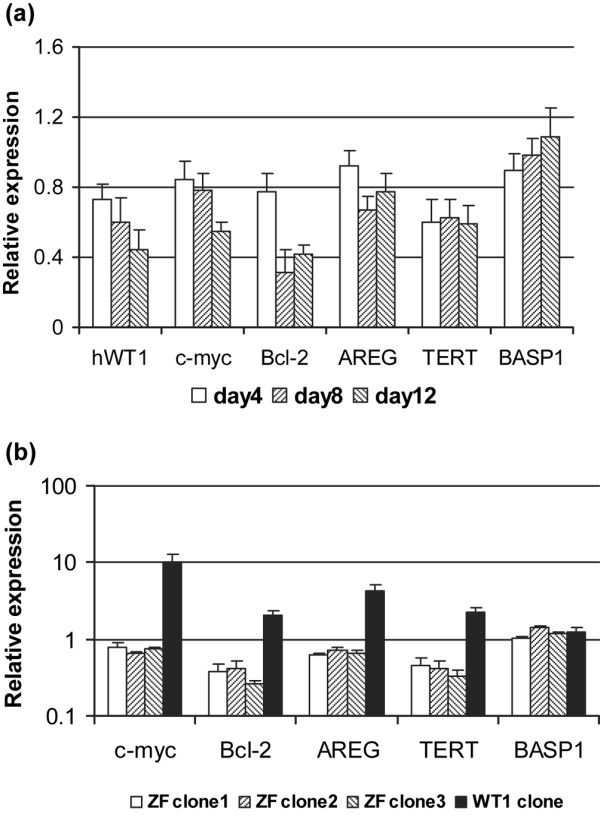
Alteration of expression of WT1 target genes in zinc finger domain lacking KTS-transfected MCF-7 cells. **(a) **Transient and **(b) **stable transfectants of MCF-7 cells were analyzed for the expression of Wilms' tumor 1 suppressor gene (WT1) target genes or nontarget genes (horizontal axis) with quantitative real-time PCR assays. The relative expression (vertical axis) of these genes in the transfected MCF-7 cells was calculated as compared with the pcDNA_3 _control. Results are the average of three experiments. AREG, amphregulin; BASP1, brain acid soluble protein 1; KTS, lysine–threonine–serine; WT1, Wilms' tumor 1 suppressor gene; ZF, zinc finger domain.

At days 4, 8 and 12, the level of WT1–ZF was increased 74-fold, 114-fold and 205-fold, respectively, as compared with control cells. In keeping with previous results, the level of the endogenous WT1 RNA progressively decreased over time in the MCF-7 cell transfectants. Similarly, the levels of the four target genes, but not BASP1, also decreased (Figure 4a). These findings were confirmed in three independent clones isolated from the transfected population (Figure 4b). In contrast, and in keeping with WT1's role as a positive regulator, the expression of these target genes was significantly upregulated in MCF-7 cells expressing full-length WT1.

### WT1–ZF - KTS inhibits c-*myc *promoter activation by full-length WT1

**Figure 5:**
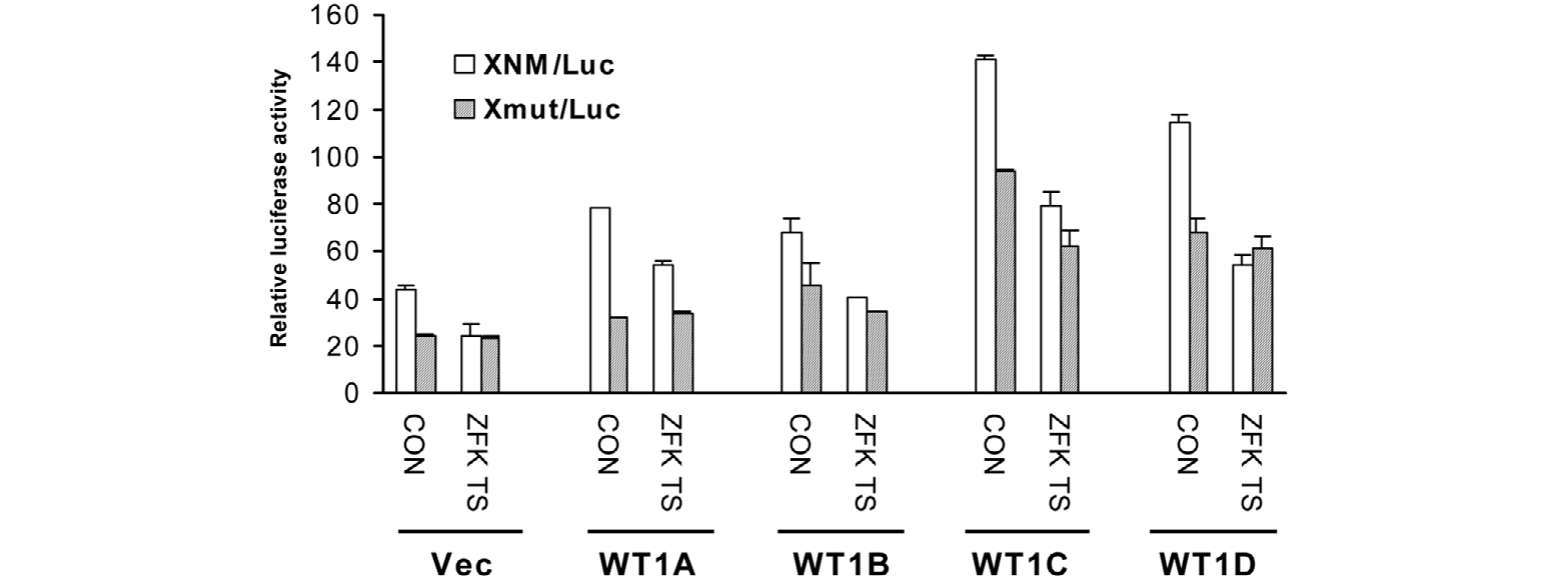
Abrogation of WT1-mediated c-*myc *transactivation by the zinc finger domain lacking KTS in MCF-7 cells. The MCF-7 cells were transiently co-transfected with the reporter constructs (either pGL2 vector, XNM/Luc or Xmut/Luc), with expression plasmids of Wilms' tumor 1 suppressor gene (WT1) isoforms (either pcDNA_3 _vector, A, B, C or D) and with WT1–zinc finger domain (ZF) (either pcDNA_3 _control (CON) or ZF lacking the lysine–threonine–serine insert (ZF-KTS)) as well as β-galactosidase expression plasmid, respectively. Relative luciferase activity (vertical axis) is indicated. Histograms show average of three independent experiments.

As a gene involved in modulating the cancer cell phenotype, WT1 has been found to regulate the expression of genes important in cell proliferation, differentiation and response to chemotherapy. We previously reported that WT1 could enhance the expression of the protooncogene c-*myc *[27]. To determine the effect of WT1–ZF on transcriptional activation of c-*myc *by full-length WT1, we co-transfected MCF-7 cells with full-length WT1, or with WT1–ZF and a c-*myc *promoter–luciferase construct that contains a single WT1 binding site (XNM/Luc). In keeping with our previous results, the WT1–ZF significantly reduced the transactivation of the c-*myc *reporter (Figure 5). This effect was not seen if the WT1 binding site was mutated (Xmut/Luc), confirming the importance of the WT1 binding site for the observed result.

### Cell growth and proliferation are inhibited by ZF - KTS

The ability of WT1–ZF to reduce/block the expression of genes such as c-*myc*, Bcl-2, AREG and WT1 itself, genes important for the survival and proliferation of breast cancer cells [34], raised the possibility that WT1–ZF could impair the growth of breast cancer cells. To assess the effect of WT1–ZF on the clonogenic capacity of cells, MCF-7 cells and MDA468 cells stably expressing ZF - KTS were plated on 10 cm dishes and their ability to form colonies was determined. The expression of ZF - KTS reduced colony formation in MCF-7 cells by 64% and that in MDA468 cells by 27%, similar to the effect observed with WT1 shRNA (Table 2). We also tested the effect of full-length WT1 and WT1–ZF in the WT1-negative breast cancer cell line MDA231. In contrast to the enhanced growth induced by full-length WT1 in MCF-7 cells and MDA468 cells, exogenous expression of WT1 produced an insignificant increase in colony formation. Furthermore, in keeping with the lack of importance of WT1 in regulating the growth of MDA231 cells, WT1–ZF did not alter their growth characteristics (Table 2).

**Table 2:** Effect of Wilms' tumor 1 suppressor gene (WT1) plasmid with ZF - KTS on the growth of breast cancer cells

Cells	pcDNA3	pcDNA3/ZF - KTS	pcDNA3/WT1	pSuper	pSuper/WT1
MCF-7					
Mean ± standard deviation (CFU)	142 ± 3.3	51 ± 4*	164 ± 9*	51 ± 4.8	40 ± 3.7*
Relative change (%)	0	-64.1	+15.5	0	-21.8
MDA468					
Mean ± standard deviation (CFU)	291 ± 8.7	214 ± 15.3*	406 ± 17*	177 ± 6.1	42 ± 1.7*
Relative change (%)	0	-26.5	+39.5	0	-76.2
MDA231					
Mean ± standard deviation (CFU)	131 ± 6.2	130 ± 3.8	142 ± 5	36 ± 1.1	32 ± 3.0
Relative change (%)	0	-0.2	+8.8	0	-11.2

Relative change represents the percentage difference in number of colony-forming units (CFU) produced in the zinc finger domain lacking the lysine–threonine–serine insert (ZF - KTS), in full-length WT1 or in WT1 shRNA transfected cells in comparison with empty vector, respectively. *P < 0.05.

**Figure 6:**
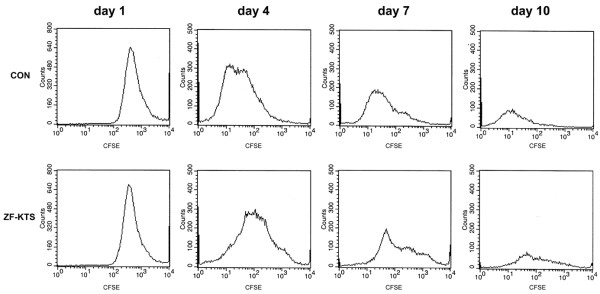
Retarded cell division in the zinc finger domain lacking lysine–threonine–serine-transfected MCF-7 cells. A typical result of three individual assays for the carboxy-fluorescein diacetate succinimidyl ester (CFSE) labeling profiles of gated transfectants of MCF-7 cells. The transfectants (CON and zinc finger domain lacking the lysine–threonine–serine insert (ZF - KTS)) and the days post G418 selection (day 1, day 4, day 7 and day 10) are indicated.

Having shown a reduced colony forming ability with ZF - KTS, we used CFSE to determine the proportion of cells undergoing one or more cell divisions over a period of time; the intensity of staining is decreased in proportion to the number of cell divisions. Control and ZF - KTS cells were labeled with CFSE and were assessed at days 1, 4, 7 and 10. The intensity was greater at all time points in the ZF - KTS-transfected cells compared with the control cells, indicating fewer cell divisions in the ZF-transfected cells (Figure 6). The number of divisions was reduced in ZF-transfected cells, as compared with control cells, by factors of 1.63, 1.85 and 2.54 at days 4, 7 and 10, respectively (Table 3). Of interest, and relevant to the above findings, was our observation that it was not possible to carry the WT1–ZF stably transfected cells for more than 3 months. In addition, transfection of ZF - KTS had no inhibitory effect on the growth of Cos-7 cells lacking WT1 expression (data not shown).

**Table 3:** Effect of the zinc finger domain lacking the lysine–threonine–serine insert (ZF - KTS) on cell division of breast cancer cells

	Mean CFSE fluorescence intensity			
	
	1 day	4 days	7 days	10 days
Control	381.82	31.25	37.92	16.80
ZF - KTS	418.21	96.89	136.32	97.65

CFSE, carboxy-fluorescein diacetate, succinimidyl ester.

### ZF - KTS induce cell death due to apoptosis

**Figure 7:**
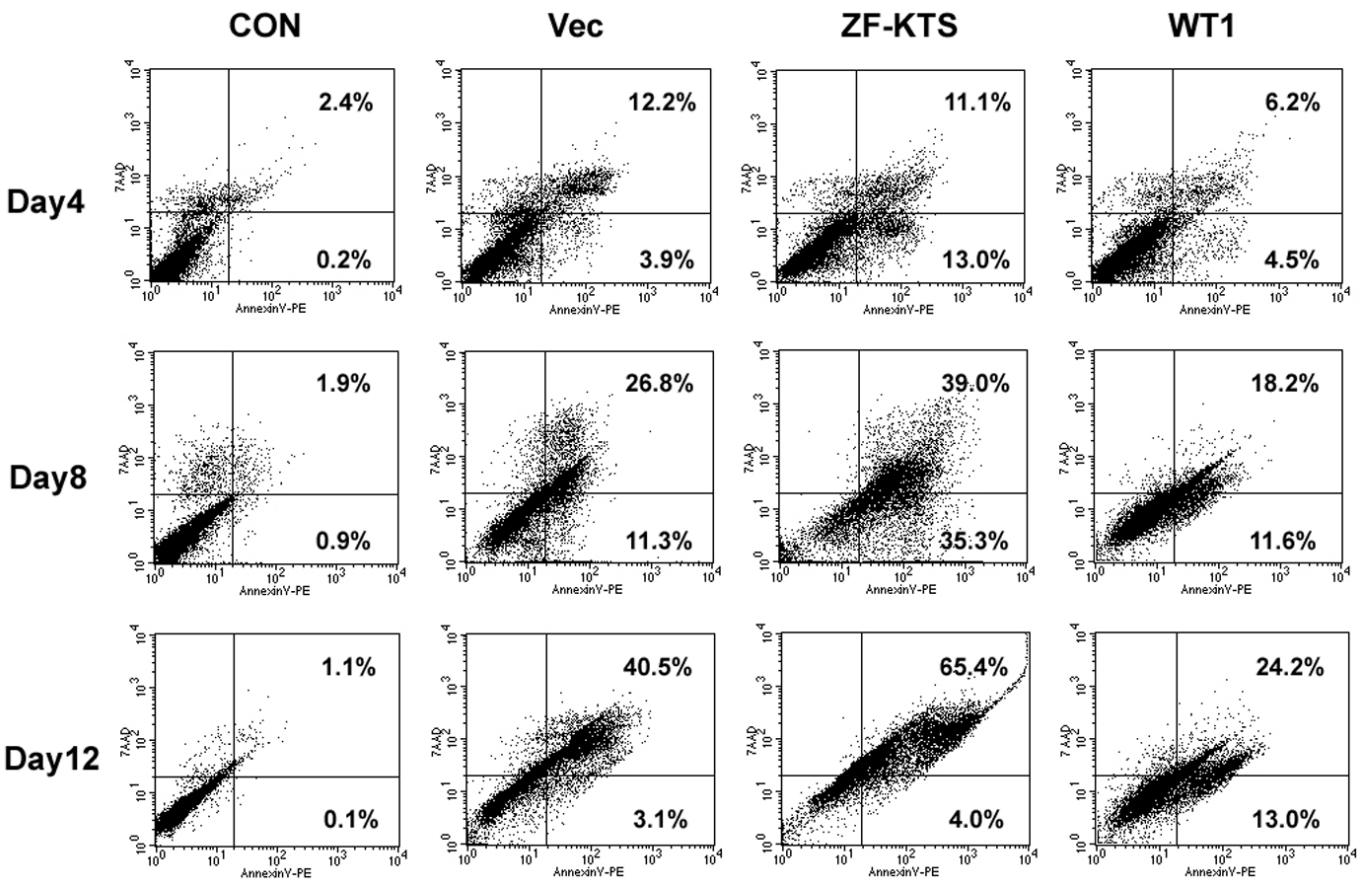
Accelerated apoptosis in the zinc finger domain lacking lysine–threonine–serine-transfected MCF-7 cells. Dot plots of a representative experiment of flow cytometric analysis for staining of Annexin V–PE (horizontal axis) and 7-amino-actinomycin D (7-AAD) (vertical axis) in nontransfected MCF-7 cells (CON) and transfected MCF-7 cells (Vec, zinc finger domain lacking the lysine–threonine–serine insert (ZF - KTS) or full-length Wilms' tumor 1 suppressor gene (WT1)). Percentages of cell populations in the different quadrants are indicated. Days after G418 selection are shown.

The reduced plating efficiency and the number of cell divisions could come about due to growth inhibition or cell death. To determine whether there is increased death in the cultures, MCF-7 cells and MCF-7–WT1–ZF cells were stained with Annexin V and 7-AAD and were assessed by flow cytometry (Figure 7). There was little evidence of apoptosis in the parental cells. Cells transfected with empty vector showed a modest increase in apoptosis, probably due to the G418 selection. The cells transfected with WT1–ZF - KTS, however, showed an increase in early apoptosis (Annexin V positive, 7-AAD negative) of 9% on day 4 and of 24% on day 8 after transfection. Similarly, late apoptosis (Annexin V positive, 7-AAD positive) was increased by 12% and by 25% on days 8 and 12, respectively. In keeping with this result, the transfection of full-length WT1 had an inhibitory effect on cell apoptosis. These results clearly demonstrate the specific nature of increased apoptosis in the WT1–ZF-expressing cells.

## Discussion

WT1 expression is important, if not critical, for the growth of some forms of cancer, and its reduced expression by antisense means or by interfering RNA can lead to the induction of differentiation, and to growth inhibition due to cell cycle arrest or cell death. For example, the induction of erythroid or megakaryocytic differentiation in K562 cells is strongly associated with decreased expression of WT1 mRNA [35]. In the present study we have explored the possible utility of WT1–ZF to block transactivation by WT1, and the consequence of this on cell behavior. In developing WT1–ZF as a tool to study the effect of WT1 on gene regulation and as a potential therapeutic target, we felt it was important to demonstrate that WT1–ZF would affect known WT1 targets. For this reason we assessed the effect of WT1–ZF on the expression of WT1 itself, and on the expression of Bcl-2 and c-*myc *in the context of WT1-expressing breast cancer cell lines. We also wanted to determine whether the WT1–ZF construct would be of value in determining the importance of the KTS insert, as our attempts at using small interfering RNA to specifically eliminate ZF - KTS or ZF + KTS in cells had been unsuccessful.

Previous studies have suggested that the N-terminal domain of WT1 functions as a dominant negative of the wild-type WT1 gene. Using reporter assays, Reddy and colleagues found that the co-transfection of N-terminal WT1 inhibited transcriptional activation mediated by WT1 in CV-1 cells, which was related to the self-association of the first 182 amino acids of WT1 [31]. This dominant-negative role of N-WT1 was further confirmed in yeast strains, and the domains involved in self-association were mapped to amino acids 1–45 and 157–253 [33]. In our reporter assays, N-WT1 had a weak suppressive effect on WT1 transcription as compared with the ZF (Figure 1). This is most probably explained by the fact that WT1–ZF inhibits by directly binding to regulatory elements in the promoter of crucial genes, while the inhibitory effect of N-WT1 requires protein–protein interactions that may be modified by post-translational modifications of the proteins or interference by other interacting proteins.

Previous studies by Rupprecht and colleagues and by Malik and colleagues demonstrated that full-length WT1 repressed the expression of WT1 promoter constructs in HEK-293 cells [25,36]. This repression was shown to be due to several WT1 binding sites between +38 and +195 of the human promoter as well as between -513 and +177 of the mouse WT1 promoter. In our studies carried out in WT1-expressing breast cancer cell lines, the co-transfection of WT1 along with reporter constructs neither inhibited nor stimulated WT1 promoter reporter expression. The lack of suppression is in contrast to what was previously observed, but is in keeping with the observations by us and other investigators that whether a particular WT1 target gene is increased or decreased in expression is dependent upon the cellular context. The failure to see enhanced expression of the WT1 promoter reporter constructs could be because the endogenous protein is already maximally stimulating the promoter or there are other, as yet unknown, cooperating factors that limit the ability of WT1 to enhance the expression of its own promoter. Using WT1–ZF we have shown that both the human and mouse WT1 promoters are negatively regulated by WT1–ZF, and that this negative regulation is due to direct binding of WT1–ZF to the promoter (Figures 1 and 2). This binding and inhibition is significant as we found decreased expression of WT1 mRNA and protein in the breast cancer cell lines transfected with WT1–ZF (Figure 3). These data clearly demonstrate that the WT1 promoter is a primary target for WT1–ZF. In keeping with this observation, we also showed that the expressions of two other WT1 targets, Bcl-2 and c-*myc*, are repressed by WT1–ZF in breast cancer cell lines.

The WT1 + KTS and WT1 - KTS isoforms occur naturally through alternate splicing of the primary transcript. Previous studies have indicated that the binding specificities of the ZF - KTS and ZF + KTS isoforms differ. For example the ZF - KTS form recognizes ERG1 sites (5'-GNGNGGGNG-3'), while the ZF + KTS form does not [37,38]. It is proposed that the insertion of the KTS tripeptide increases the flexibility between ZF3 and ZF4, thus disrupting the binding of ZF4 to its cognate sites in the DNA major groove [39]. This is illustrated by the reduced number of complexes formed between the mouse WT1 promoter with ZF + KTS as compared with the ZF - KTS form of WT1 [25]. In our DNA binding assays we also found that the ZF - KTS form bound to most WT1 sites in the WT1 promoter, while the ZF + KTS isoform only bound to sites between nucleotides -212 and -21 (Figure 2). This is consistent with previous studies showing that the ZF + KTS form of WT1 binds nucleotides -171 to -157 and nucleotides -155 to -141 in the human promoter, and binds nucleotides -203 to -192 in the mouse promoter [25,28]. In our studies we found that ZF - KTS was a stronger repressor of the WT1 reporter than the ZF + KTS form. This may be due to more binding of the ZF - KTS protein to the promoter region, or it may reflect a greater importance of the more upstream binding sites. Regardless, for all reporters tested, it appears that ZF - KTS is more potent at blocking transcription than the ZF + KTS isoform.

Consistent with the ability to suppress the expression of genes important for cell growth and survival, transfection of WT1–ZF - KTS into the breast cancer cell line MCF-7 had a marked effect on cell behavior. Consistent with reduced expression of c-*myc*, the cells entered the cell cycle less often and at a slower rate than the parental cells. In keeping with the reduced levels of Bcl-2, there was increased apoptosis as measured by Annexin V/7-AAD staining. Finally, in keeping with the reduced expression of TERT, there was a reduction in plating efficiency over time – and eventually the cells ceased growing altogether.

## Conclusion

In this manuscript we show that WT1–ZF can behave as a dominant negative of WT1 in WT1-expressing breast cancer cells. These studies further confirm the importance of WT1 in the growth of some forms of breast cancer, and demonstrate the importance of WT1 in regulating apoptosis, the cell cycle and self-renewal capacity. The WT1–ZF construct should prove to be an important reagent in identifying genes and cellular processes regulated by WT1. Finally, the inhibitory effect of WT1–ZF - KTS on breast cancer growth supports the development of such a molecule or other inhibitors of WT1 in the control of breast cancer and other malignancies characterized by WT1 overexpression.

## Abbreviations

7-AAD = 7-amino-actinomycin D; ALDH = aldehyde dehydrogenase 1 family member A2; AREG = amphregulin; BASP1 = brain acid soluble protein 1; CFSE = carboxy-fluorescein diacetate succinimidyl ester; HA = heme agglutinin; KTS = lysine–threonine–serine; PCR = polymerase chain reaction; RT = reverse transcriptase; SDS = sodium dodecyl sulfate; shRNA =short hairpin; RNA; TERT = human telomerase reverse transcriptase; WT1 = Wilms' tumor 1 suppressor gene; ZF = zinc finger domain; ZF-KTS = zinc finger domain protein lacking lysine–threonine–serine of WT1; ZF+KTS = zinc finger domain protein with lysine–threonine–serine of WT1.

## Competing interests

The authors declare that they have no competing interests.

## Authors' contributions

YH designed the study, performed the experiments and drafted the manuscript. SS-M and HK participated in data analysis and modified the manuscript. LY participated in the data analysis. MDM reviewed and modified the manuscript.
